# Insights Into Central Congenital Hypothyroidism: A Multicenter Retrospective Analysis

**DOI:** 10.1210/clinem/dgae485

**Published:** 2024-07-15

**Authors:** Alina German, Shlomo Almashanu, Liat de Vries, Merav Gil Margolis, Rana Halloun, Alon Haim, Ori Eyal, Floris Levy-Khademi, Dikla Pivko-Levy, Judith Nir, Orit Pinhas-Hamiel, Yardena Tenenbaum-Rakover

**Affiliations:** Pediatric Endocrinology and Diabetes Unit, Ha’Emek Medical Center, Afula 1834111, Israel; The National Newborn Screening Program, Ministry of Health, Tel Hashomer, Ramat Gan 5262000, Israel; The Jesse Z and Sara Lea Shafer Institute for Endocrinology and Diabetes, National Center for Childhood Diabetes, Schneider Children's Medical Center of Israel, Faculty of Medicine, Tel-Aviv University, Tel Aviv 6997801, Israel; The Jesse Z and Sara Lea Shafer Institute for Endocrinology and Diabetes, National Center for Childhood Diabetes, Schneider Children's Medical Center of Israel, Faculty of Medicine, Tel-Aviv University, Tel Aviv 6997801, Israel; Pediatric Endocrinology Unit, Ruth Rappaport Children's Hospital, Haifa 3109601, Israel; Pediatric Endocrinology and Diabetes Unit, Soroka Medical Center, Faculty of Health Science Ben-Gurion University of the Negev, Beer-Sheva 8410101, Israel; Pediatric Endocrinology Unit, Dana-Dwek Children's Hospital, Tel-Aviv Medical Center, Faculty of Medicine, Tel-Aviv University, Tel Aviv 6997801, Israel; Pediatric Endocrinology and Diabetes Unit, Share Zedek Medical Center, Faculty of Medicine Hebrew University, Jerusalem 9103102, Israel; Pediatric Endocrinology and Diabetes Unit, Wolfson Medical Center, Holon 5822012, Israel; Pediatric Endocrinology and Diabetes Unit, Shamir (Asaf Harofe) Medical Center, Zerifin 7035101, Israel; Pediatric Endocrine and Diabetes Unit, Edmond and Lily Safra Children's Hospital, Sheba Medical Center, Ramat Gan 5224213, Israel; Consulting Endocrine Unit, Clalit Health Services, Afula 1834111, Israel

**Keywords:** central congenital hypothyroidism, CCH, neonatal screening program, NSP, total T4, TT4, free T4, FT4, thyroid-stimulating hormone, TSH

## Abstract

**Context:**

Central congenital hypothyroidism (CCH) is a thyroid hormone deficiency at birth caused by inadequate pituitary stimulation of the thyroid gland. Although primary congenital hypothyroidism has been studied extensively, studies on CCH are sparse.

**Objective:**

To assess the prevalence of CCH in Israel and describe its clinical features, neonatal screening results, and outcomes.

**Methods:**

This multicenter cross-sectional retrospective chart review covered 9 pediatric endocrine units throughout Israel; patients diagnosed with CCH in 1987–2021 were categorized into early (within 14 days of life) and late (after 14 days) diagnosis groups. Newborn screening (NBS) results were retrospectively retrieved from the national NBS program dataset.

**Results:**

CCH prevalence in Israel was about 1:42 800 live births. Subjects were 94 patients (54 males), of these, 84% had multiple pituitary hormone deficiencies and 16% had isolated CCH. The median age at diagnosis was 50 days (range, 1-8760), with 66% having moderate to severe hypothyroidism. NBS detected only 3 infants. Early diagnosis occurred in 34% due to hypopituitarism, while 66% were diagnosed later due to growth and developmental delays. Neurodevelopmental sequelae included mental retardation (12%), learning difficulties (18%), delayed speech (27%), and motor clumsiness (19%), with no significant differences in outcomes between early and late diagnosis.

**Conclusion:**

Despite high rates of neurodevelopmental sequelae, no differences were found between early and late diagnosis groups. Further research is needed to assess the impact of delayed diagnosis on neurological outcomes in newborns with CCH. Improved strategies for detecting CCH in newborns are also necessary.

Central congenital hypothyroidism (CCH) is a rare disorder that occurs due to insufficient hypothalamic-pituitary stimulation of an otherwise normal thyroid gland (1, 2). It is characterized by low total T4 (TT4) with either low, normal or slightly elevated thyroid-stimulating hormone (TSH) levels.

TSH deficiency in CCH is either isolated, mainly due to specific gene mutations, or occurs with coexisting multiple pituitary hormone deficiencies (MPHD) (3). In isolated CCH (ICCH), the specific gene mutations reported include inactivation of the TSH β-subunit, the thyrotropin-releasing hormone receptor (*TRHR*), *TBL1X*, *IRS4*, and *IGSF1* (3, 4). In MPHD, the genetic background was reported in 30% of patients and included mutations in *POU1F1*, *PROP1*, *HESEX1*, *LHX3*, *LHX4*, and others (1-3).

Thyroid hormones are critical for normal brain development in early life (4). As a result, hypothyroidism can lead to irreversible cerebral damage. Likewise, a delay in the diagnosis and initiation of levothyroxine (LT4) supplemental therapy may result in neurological and intellectual sequelae (5-8). The common approach for assessing thyroid function in newborn screening (NBS) programs worldwide is by measuring first-line TSH levels followed by measuring TT4 when TSH is elevated. Implementation of such NBS programs worldwide allowed identification and treatment of congenital hypothyroidism (CH) before clinical recognition, leading to the virtual disappearance of the intellectual disabilities that were found in up to 28% of affected children in the pre-NBS era (9). However, while this approach successfully detects newborns with primary CH, it often fails to identify those with CCH, since TSH levels in these infants are either low, normal, or mildly elevated. This prompted several countries around the world to modify their NBS strategy, opting to initially measure TT4 or free T4 (FT4), with TSH measured simultaneously or immediately afterward if necessary. This new strategy allows the detection of neonates with CCH (10, 11). In the Netherlands, the estimated prevalence of CCH increased from 1:106 304 in the late 1970s to 1:13 000 in the last 2 decades following the introduction of the new strategy (3).

The thyroid NBS program implemented in Israel since 1978 uses TT4 as a screening test, and TSH is measured only when TT4 falls within the lowest 10th percentile. Unfortunately, the common thyroxine-binding globulin (TBG) deficiency also causes low TT4 in combination with normal TSH, resulting in a substantial number of false-positive referrals (12). Therefore, when TSH is not elevated, the results are not reported to the referring physicians. This means that newborns with CCH may be missed, leading to delay in the diagnosis of these infants.

In the current study, we performed a nationwide, multicenter cross-sectional review of patients diagnosed with CCH over a period of 33 years in Israel. Our objectives were to assess the prevalence of CCH in Israel and to report on the clinical and endocrine characteristics and long-term outcomes of infants with CCH.

## Methods

A multicenter, nationwide cross-sectional retrospective study was conducted on patients diagnosed with CCH between January 1987 and December 2021. Included were all patients diagnosed with CCH over that period in 9 pediatric endocrine units in referral medical centers in Israel. We excluded patients diagnosed with primary CH and patients with acquired primary hypothyroidism or central hypothyroidism due to causes such as brain tumor, radiation, surgery, trauma, or central nervous system infection.

Data included demographic, family, and birth history; reason for the referral for endocrine evaluation, including the presence of jaundice; hypoglycemia (defined as serum glucose level below 50 mg/dL); clinical findings, including physical examination, height, and weight measurements at first visit; endocrine parameters, results of stimulation tests; brain imaging and molecular results where available; neurodevelopmental evaluation; and growth parameters at the last visit.

The cohort was divided into early and late groups based on age at diagnosis. Specifically, following the 2020–2021 consensus guidelines update for CH (13), patients were categorized into the early group if the diagnosis of CH was confirmed within 14 days of life, and into the late diagnosis group if the diagnosis was established thereafter. NBS results for infants with a CCH diagnosis were compared between the early and late groups.

### Biochemical Parameters

Thyroid function test results are consistent with the diagnosis of CCH were defined as a low FT4 with inappropriately normal TSH levels. This diagnosis was also made in patients with mild or moderately elevated TSH (> 6 and < 20 mU/L) after exclusion of other forms of hypothyroidism. The FT4 reference range provided by the laboratory was 11 to 22 pmol/L, and for newborns, the normal range was higher, 10 to 26.8 pmol/L. The TSH reference range provided by the laboratory was 0.2 to 4.0 mIU/L, and for newborns, it was 0.4 to 10 mIU/L. Subjects with FT4 results below 10 pmol/L at a confirmation test were defined as having moderate to severe CCH, according to guidelines of the European Society for Pediatric Endocrinology (13).

All patients were examined by a pediatric endocrinologist and underwent evaluation for anterior pituitary hormone deficiencies. Growth hormone (GH) deficiency was diagnosed by a GH response less than 7.5 ng/mL after 2010/2012, or less than 10.0 ng/mL before that, following 2 different GH stimulation tests, in line with the local guidelines for GH deficiency diagnosis. Adrenocorticotropic hormone (ACTH) deficiency was diagnosed by peak cortisol less than 500 nmol/L following an ACTH stimulation test. Gonadotropin-releasing hormone (GnRH) deficiency was defined by a lack of pubertal signs at the appropriate age (> 13 years in females and > 14 years in males) or failure to progress through puberty, along with a luteinizing hormone (LH) response less than 5 IU/L following a GnRH stimulation test.

### Imaging

Brain magnetic resonance imaging (MRI) was performed in all patients with MPHD and in some patients with ICCH. The results were reviewed by an experienced neuroradiologist.

### Neurodevelopmental Evaluation

In the study, information on developmental delay was obtained from the patients’ medical charts and was based on reports by a developmental pediatrician or pediatric neurologist and/or therapeutic intervention in neurologic or child development departments. All children in Israel from birth to the age of 6 years undergo these regular assessments (14), which check personal–social, language, and fine and gross motor skills using structured milestone attainment checklists. Children who fail to meet expected developmental milestones are referred for further evaluation by a developmental pediatrician or neurologist.

### Neonatal Screening

Blood samples were collected by heel puncture 48 to 72 hours after birth. Between 1987 and 2006, the tests were performed using Diagnostic Products Corp. (Los Angeles, CA) radioimmunoassay TT4 and TSH kits. Since 2006, they have used the Perkin Elmer B065-112 AutoDELFIA neonatal TT4 and B032-312 AutoDELFIA neonatal TSH kits, both of which employ time-resolved fluoro-immunoassay (PerkinElmer Life and Analytical Sciences, Wallac Oy, Turku, Finland).

The Israeli neonatal screening program is based on the TT4 level, followed by a confirmatory TSH test when TT4 is below the 10th percentile. Until 2006, all low-screening TT4 results were reported, regardless of whether TSH levels were elevated. Since 2006, only TSH values above 20 mIU/L, consistent with primary CH, are reported. Results are reported to the pediatric endocrinologist in charge of the geographical area.

Computerized records were available from 2008. Hence, neonatal screening results were retrospectively retrieved from the computerized database for infants detected in the multicenter study and born between January 2008 and December 2021.

### Molecular Analysis

Molecular analysis was available for 23 patients (24%). The genetic testing modality used was either target gene analysis, whole exome sequencing (WES), or panel next-generation sequencing (NGS).

### Statistical Analysis

SPSS 16.0 (SPSS Inc., Chicago, IL) was used for statistical analysis. Descriptive statistics were computed for all variables. The Mann–Whitney *U* test was used to compare continuous variables. The chi-square test was used to compare categorical variables. Considering that we have a relatively small sample number, Fisher's exact test was performed for categorical variables. Two-tailed tests were performed for all analyses. *P* < .05 was considered statistically significant.

### Ethics Approval

This study was performed in line with the principles of the Declaration of Helsinki. Approval was granted by the institutional review boards of all involved medical centers. Informed consent was waived as subjects were not identified.

## Results

A total of 94 patients (54 male) were enrolled in the study. Demographic characteristics of the patients are presented in Table 1. Seventy-nine patients (84%) had MPHD and 15 (16%) had ICCH. Of the latter group, 80% were male (*P* = .05). Only 3 infants (9%) from the whole cohort were detected by the NBS program.

**Table 1.** Demographic and perinatal data of patients with central congenital hypothyroidism

	All	MPHD	ICCH	*P* value
No. (%)	94 (100)	79 (84)	15 (16)	
Male (%)	54 (57)	42 (53)	12 (80)	**.05**
Consanguinity (%)	21 (22)	19 (24.4)	2 (12.3)	.3
Other cases in the family (%)	13 (14.5)	5 (6.4)	8 (53.3)	**.001**
Gestational age, weeks	38.8 ± 2.9	38.6 ± 3.1	36.6 ± 4.8	NS
Birth weight, SDS	−0.49 ± 1.2	−0.6 ± 1.2	0.1 ± 1.4	NS
Age at diagnosis, days	50 (1-8760)	55.5 (2-8660)	45.5 (1-2190)	NS
Early diagnosis before 14 days	28 (30)	24 (30)	4 (27)	.80
Late diagnosis after 14 days	66 (70)	55 (70)	11 (73)	.81

Numbers represent n (%), median (range), or mean (±SD). *P* values below .05 are shown in bold type.

Abbreviations: ICCH, isolated congenital central hypothyroidism; MPHD, multiple pituitary hormone deficiency; SDS, standard deviation score.

Consanguinity was reported in 21 patients (22%). Five patients (6.4%) in the MPHD group and 8 (53%) in the ICCH group had additional family members with CCH (*P* < .001).

The mean gestational age at birth for the entire cohort was 38.8 ± 2.9 weeks, with mean birth weight SDS of −0.5 ± 1.2. These figures were comparable between the MPHD and ICCH groups.

Initial endocrine evaluation was performed at a median age of 50 days (range, 1-8760 days). Twenty-eight patients (30%) were diagnosed with CCH during their first 14 days of life, while 66 (70%) were diagnosed later. The reasons for referral among the subjects with early diagnosis are summarized in Table 2. Among the early diagnosis group, 24 infants (86%) had MPHD and presented with common signs and symptoms of hypopituitarism, including hypoglycemia (92%), prolonged jaundice (33%), and micropenis (33% of males). Patients who presented with hypoglycemia were diagnosed at a median age of 7 days (range, 1-14). All were included in the early diagnosis group.

**Table 2.** Early diagnosis group

Reason for referral	MPHD	ICCH	*P* value
No. (% out of the early group; n = 28)	24 (86)	4 (14)	**<**.**0001**
Hypoglycemia	22 (92)	0	.**004**
Prolonged jaundice	8 (33)	2 (50)	.51
Additional cases in the family	3 (12.5)	3 (75)	.**005**
Micropenis	5 (33% of males)	0	NS
NBS reported*^a^*	2 (0.8)	1 (25)	.**003**
Others: Maternal hypothyroidism, choanal atresia	3 (12.5)	0	

Clinical presentation of patients with MPHD and ICCH. Numbers represent n (%). Numbers represent n (%). *P* values below .05 are shown in bold type.

Abbreviations: ICCH, isolated congenital central hypothyroidism; MPHD, multiple pituitary hormone deficiency; NBS, Newborn screening program.

^a^For infants born before 2008.

Four subjects (14%) from the early diagnosis group had ICCH, of whom 3 patients were referred because of previous diagnosis of ICCH in siblings. One of these 3 patients also had prolonged neonatal jaundice.

In the late diagnosis group, 55 patients (83%) had MPHD and presented with poor growth (56%), prolonged jaundice (18%), and developmental delay (7%) (Table 3). Eleven subjects (17%) in the late diagnosis group had ICCH, comprising 73% of the ICCH cohort. Most of them (72%) were male and were referred for prolonged jaundice (18%), macroglossia (9%), coarse voice (9%), and developmental delay (9%).

**Table 3.** Late diagnosis group

Reasons for referral	MPHD	ICCH	*P* value
No. (% out of the late group; n = 66)	55 (83)	11 (17)	**<**.**001**
Male	27 (40)	8 (72)	.**05**
Failure to thrive	8 (14)	0	
Prolonged jaundice	10 (18)	2 (18)	1.0
Macroglossia	0	1 (9)	
Coarse voice	0	1 (9)	
Poor growth	23 (42)	1 (9)	.**03**
Developmental delay	4 (7)	1 (9)	.81
Delayed puberty	2(3)	0	

Clinical presentation of patients with MPHD and ICCH. Numbers represent n (%). *P* values below .05 are shown in bold type.

Abbreviations: NBS, Newborn screening program; MPHD, multiple pituitary hormone deficiency; ICCH, isolated congenital central hypothyroidism.

Endocrine findings are presented in Table 3. NBS endocrine results were available for 44 subjects born after 2008 (Table 4). Among infants diagnosed with CCH, mean screening TSH level was 4.5 ± 3.1 mIU/L (range, 0-14), and mean TT4 for the term neonates was 7.0 ± 2.8 µg/dL (range, 1-14). Patients with MPHD had mean screening TSH of 4.2 ± 2.8 mIU/L and mean TT4 of 6.8 ± 3.1 µg/dL, while patients with ICCH had TSH of 7.1 ± 4.5 mIU/L and TT4 of 5.3 ± 1.8 µg/dL. These latter results were comparable between the 2 groups. Six infants (13.6%), all diagnosed with MPHD, had TT4 levels above the NBS cutoff value of the 10th centile.

**Table 4.** Results of the newborn screening program for TSH and TT4

NBS	All n = 44	MPHD n = 32	ICCH n = 12	Normal range	*P* value
TSH, mIU/L	4.5 ± 3.1	4.2 ± 2.8	7.1 ± 4.5	< 20	.13
TSH range, mIU/L	(0-14)	(0-11)	(1-14)		
TT4, μg/dL	7.0 ± 2.8	6.8 ± 3.4	5.3 ± 1.8	< 10%*^a^*	.09
TT4 range, μg/dL	(1-14)	(1-14)	(3-9)		

Screening TSH and TT4 are presented as mean ± SD. Numbers represent n (%).

^*a*^TT4 results lower than the 10th centile are considered as abnormal.

Mean confirmatory laboratory TSH and FT4 levels were 3.7 ± 2.7 mIU/L and 8.3 ± 2.7 pmol/L, respectively (Table 5). Sixty-three patients (67%) had moderate to severe CCH at diagnosis. In addition to CCH, patients with MPHD had GH deficiency (96%), ACTH deficiency (73%), and diabetes insipidus (5%).

**Table 5.** Confirmation of laboratory results for TSH and FT4 at the time of diagnosis for the study cohort

	All n = 94	MPHD n = 79	ICCH n = 15	Normal range	*P* value
Laboratory TSH, mIU/L	3.7 ± 2.7	3.4 ± 2.3	5.24 ± 4.2	0.4-4.2	.21
Laboratory FT4, nmol/L	8.3 ± 2.7	8.1 ± 2.9	8.8 ± 2.0	11-22	.43
Laboratory FT4, nmol/L	8.3 ± 2.7	8.1 ± 2.9	8.8 ± 2.0	11-22	.43
FT4 < 10, nmol/L	63 (67)	54 (68)	9 (60)		.55
GHD		76 (96)			
ACTH deficiency		58 (73)			
Diabetes insipidus		4 (5)			
Abnormal MRI imaging		68 (92)			

Laboratory TSH and FT4 results are presented as mean ± SD. Numbers represent n (%).

Abbreviations: ACTH, adrenocorticotropic hormone; GHD, growth hormone deficiency; MRI, magnetic resonance imaging.

### Imaging

CNS imaging was conducted on all 79 subjects diagnosed with MPHD. MRI results were available for 74 subjects and were abnormal in 68 (92%). Abnormal findings included pituitary stalk interruption syndrome (PSIS; characterized by the triad of thin or interrupted pituitary stalk, absent or ectopic posterior lobe, and hypoplastic and aplastic anterior lobe) in 45 (63%), pituitary hypoplasia in 21 (25%), and empty sella syndrome in 2 patients (3%). Two patients had additional brain anomalies, including brain cysts, cortical hypoplasia, and agenesis of the corpus callosum.

### Molecular Results

Molecular analysis was performed in 16 subjects with MPHD. Pathogenic mutations identified included *POU1F*1 in 5 patients, *HESX1* in 3, *GLI3*, *MAD*, *PROK2*, and *TTC26* in 1 patient each, and maternal inherited translocation 46XXt (14, 17) in 1 subject. Seven subjects with ICCH underwent molecular analysis. Those results revealed 6 with X-linked *IGSF1* gene mutations and 1 with an autosomal dominant *CHD7* mutation.

### Neurodevelopmental Outcome

Neurologic sequelae were reported in 34 subjects (37%) and included delayed speech, motor clumsiness, learning difficulties, and mental retardation. Neurologic sequelae were more frequent among patients from the ICCH group than the MPHD group (60% vs 32%, *P* = .04). The 2 groups did not differ in specific neurologic functions (Table 6). Three subjects with MPHD from the early diagnosis group who developed mental retardation were diagnosed in the first 2 weeks of life due to severe hypoglycemic episodes. The frequency of delayed diagnosis between the 2 groups was similar. No differences were found in neurodevelopmental sequelae between patients diagnosed early (before 14 days of life) and those diagnosed late (Table 7).

**Table 6.** Neurodevelopmental sequelae in patients with CCH

	Total	MPHD	ICCH	*P* value
No.	92	77	15	
Age at diagnosis, days: median, range	50.0 (1-8760)	50.5 (2-8660)	45.5 (1-2190)	.65
Late diagnosis	66 (72)	55 (71)	11 (73)	.87
Any neurological sequelae	34 (37)	25 (32)	9 (60)	.04
Delayed speech	25 (27)	19 (25)	6 (40)	.22
Motor clumsiness	18 (19)	15 (19)	3 (20)	.90
Learning difficulties	17 (18)	14 (18)	3 (20)	.85
Mental retardation	11 (12)	10 (13)	1 (7)	.51

Comparison between patients with MPHD and patients with ICCH. Numbers represent n (%).

Abbreviations: CCH, central congenital hypothyroidism; ICCH, isolated central congenital hypothyroidism; MPHD, multiple pituitary hormone deficiency.

**Table 7.** Neurodevelopmental sequelae in patients with CCH

	Early n = 28	Late n = 64	*P* value
MPHD/ICCH (%)	24/4 (86/14)	53/11 (83/17)	
Moderate to severe CCH FT4 < 10 (nmol/L)	22 (78)	41 (64)	.18
Any neurological sequelae	7 (25)	25 (39)	.19
Delayed speech	7 (25)	19 (30)	.62
Motor clumsiness	4 (14)	14 (22)	.37
Learning difficulties	3 (11)	16 (25)	.13
Mental retardation	2 (7)	9 (14)	.34

Comparison between patients diagnosed before the age of 14 days (early group) and patients diagnosed after the age of 14 days (late group). Numbers represent n (%).

Two patients from the MPHD group had brain anomalies and were not included in the analyses.

Abbreviations: CCH, central congenital hypothyroidism; ICCH, isolated central congenital hypothyroidism; MPHD, multiple pituitary hormone deficiency.

### Prevalence of CCH

Among 2 301 315 live infants born between January 2008 and December 2021 in Israel, 1 885 075 were born at the hospitals participating in this study. Of those infants, 44 patients were diagnosed with CCH in the same period, resulting in an estimated prevalence of 1:42 842 in Israel.

### Newborn Screening Thresholds

We explored whether modifying the NBS reporting protocol to incorporate a lower threshold of TT4 levels, specifically to 6 µg/dL or 7 µg/dL would improve diagnosis of CCH without increasing the number of false-positive results. Annually, approximately 200 000 infants are born in Israel, with around 1000 newborns exhibiting TT4 levels below 6 µg/dL. From 2008 to 2021, 2 301 315 babies were born, and approximately 14 000 had TT4 levels below 6 µg/dL. In our study cohort, 26 (59%) subjects had screening TT4 levels below 6 µg/dL, indicating that with that threshold, 41% of newborns with CCH would be missed. Using a threshold of 7 µg/dL, around 2000 newborns per year will fall below this level. In our study, 30 (68%) newborns had TT4 below 7 µg/dL, indicating that with that threshold, 32% of newborns with CCH would be missed.

Considering the estimated prevalence of CCH in Israel at 1:42 842, incorporating a lower threshold for TT4 would lead to a substantial number of false-positive results with a high rate of missing CCH diagnosis.

## Discussion

This study considers the evaluation of patients diagnosed with CCH over a period of 33 years in 9 pediatric endocrinology centers in Israel. We demonstrate that the majority of patients with CCH were diagnosed late, with initial endocrine evaluation performed only at a median age of 50 days (range, 1-8760 days), and only 3 infants were identified by NBS. More than half had moderate to severe hypothyroidism at diagnosis. Our results indicate that although a newborn screening program based on initially measuring total T4 followed by TSH theoretically allows the detection of neonates with CCH, the current screening and reporting protocol in Israel, which only flags low TT4 levels when TSH is elevated, still results in missing newborns with CCH. We also found a high rate of coexisting hormonal deficiencies in MPHD subjects, especially GH (96%) and ACTH (73%), which add to the risk of brain-damaging hypoglycemia and circulatory shock and highlight the importance of early diagnosis of such children. In addition, we found a high rate of neurologic sequelae (37%). Although previous research suggested that delayed diagnosis may influence the frequency of neurological sequelae (6), we found no differences in this respect between the early and late diagnosis groups. However, this conclusion should be drawn with caution, as the lack of difference might be attributed to the small size of the early diagnosis group.

Our results corroborate prior studies indicating that most patients diagnosed with CCH have MPHD (84% in the present study). Additionally, consistent with earlier reports (6, 15, 16), the MPHD group displayed GH deficiencies (96%), ACTH deficiencies (73%), and diabetes insipidus (5%). Only one-third of these patients were diagnosed early, with hypoglycemia being the predominant presenting symptom (92%), followed by neonatal jaundice and/or micropenis in male patients. Importantly, despite the existence of additional hormonal deficiencies, two-thirds of the patients were diagnosed late, upon manifestations of growth failure and developmental delay. Our findings support previous reports (17) indicating that over half of patients with CCH (64% in the present study) presented with moderate to severe hypothyroidism at the time of diagnosis. Naafs et al reported that most neonates with MPHD were only diagnosed after notification of an abnormal NBS result, even though many patients were hospitalized in the first weeks of life for either feeding problems, hypoglycemia, or prolonged jaundice (18).

Abnormal MRI findings were observed in 92% of individuals diagnosed with MPHD. In our study population, pituitary stalk interruption syndrome (PSIS) was the predominant pituitary anomaly, affecting 63% of patients. This is in keeping with previous findings that PSIS is the most common pituitary malformation among MPHD patients (3).

Sixteen percent of the cohort had ICCH. Most were diagnosed late, similar to MPHD subjects, often with other cases present within their families. These findings are consistent with earlier reports (19). Diagnosis of ICCH is often delayed because other pituitary hormone secretions are not impaired, and symptoms of hypothyroidism may appear later. However, in jurisdictions whose NBS programs incorporate TSH and FT4/T4 measurements, the prevalence of ICCH was shown to be higher, ranging from 33% to 40% of patients with CCH (15, 18, 20, 21).

The estimated prevalence of CCH in the current study is 1:42 842 live births, lower than other worldwide reports. The reported prevalence in the Netherlands is 1.5 times higher, at 1:25 642 (18). In Japan, it is 1:30 833 (20), and in the northwest states of the United States it is 1:60 269 (22). The lower prevalence in our study relative to the Netherlands and Japan may be due to incomplete enrollment and underdiagnosis of cases with ICCH. The traditional approach used in NBS programs worldwide for screening thyroid function—first measuring TSH, followed by TT4 when TSH is elevated—focuses on diagnosing primary CH and misses CCH. By contrast, TT4-based NBS has the advantage of identifying newborns with CCH who have low TT4 without elevated TSH. Yet despite the use of this strategy by the Israeli NBS program, only 3 newborns in our cohort were identified, indicating a gap in effectively capturing cases of CCH. To avoid missing infants with CCH, Japan simultaneously measures TSH and FT4 in all neonates, yielding screening sensitivity of only 59.1% (20). A study in northeastern Italy, where NBS simultaneously measured TT4 and TSH, found that 10 neonates with CCH were missed over 13 years; all had TT4 levels above the cutoff of 40 nmol/L (16). Similarly, among 47 patients in Indiana diagnosed with CCH, 81% initially had normal TT4 levels, and LT4 initiation was significantly delayed (6). Those authors concluded that most children with CCH have normal thyroid function at birth, meaning that measuring TSH and TT4 simultaneously still misses the majority of newborns with CCH. Indeed, in our cohort, 7 infants (14%) had screening TT4 levels above 10 µg/dL. Considering a lower TT4 cutoff value may reduce false-positive results but will miss higher rates of newborns with CCH. To avoid these pitfalls, NBS in the Netherlands consists of primary T4, followed by TSH measurement for those in the lowest 20% of T4 concentrations, and TBG measurement for those in the lowest 5% of TT4. This enables the calculation of the T4/TBG ratio, providing an estimation of the FT4 concentration. This 3-step approach effectively detects primary CH and CCH (18) and may explain the high reported prevalence of CCH in the Netherlands mentioned above. Therefore, we propose that enhancing CCH detection requires careful refinement of the NBS protocol.

A high rate of neurodevelopmental sequelae was found in our cohort (37%), including mental retardation, learning disorders, delay in language acquisition, and motor clumsiness. These were more common in the ICCH group (*P* = .04). No significant differences in neurologic sequelae were shown between early and late diagnosis of CCH. However, the rate of neurological sequelae was substantially lower in the early diagnosis group (25% compared to 39%). This suggests that the observed lack of significance could be attributed to the limited size of this group. Additionally, while a 14-day cutoff to differentiate between early and late diagnosis groups is the standard of care, 14 days may be too early to detect clinically significant differences in neurodevelopmental outcomes.

Developmental delay in patients with CCH can be attributed to syndromes involved in cerebral abnormalities like midline brain defects, or to mutations in genes like *IGSF1* or *CHD7* that are associated with neurologic involvement (23). Indeed, we have shown previously that patients with *IGSF1* mutations presented with motor clumsiness, mild psychomotor delays, and speech delay, attributed possibly to the involvement of *IGSF1* in brain development (24). Hypoglycemic events in MPHD are another contributor to neurologic sequelae. Only a few reports have compared outcomes among newborns with CCH in association with the time at which supplemental LT4 therapy is initiated. Nebesio et al reported the neurologic outcomes of 42 newborns with CCH and showed that 50% exhibited developmental delay (6). Medical charts were used to assign developmental delay, which was based on parental reports, visits to a developmental pediatrician, or involvement in early intervention therapies. When comparing patients detected through neonatal screening with those diagnosed clinically later in life, developmental delay was observed in 25% of the early-detection group, compared with 56% of the late-detection group. Our results are consistent with those reported by Naafs et al (25), who examined 87 patients with early-treated CCH alongside their siblings, focusing on the full-scale intelligence quotient (FSIQ) and motor function as secondary outcomes. They found that 32% of MPHD subjects exhibited an FSIQ below 85, compared to 15% in siblings, and 9% scored an FSIQ below 70, unlike any of the sibling controls (*P* = .04). Delayed speech was not a target measure in that study. However, in our cohort of ICCH, 46% (7 subjects) demonstrated delayed speech, thereby increasing the overall proportion of ICCH patients with any developmental sequelae. This pattern was notably observed in patients with late-diagnosed *IGSF-1* mutations (19). Considering motor development, our findings are consistent with those reported by Naafs et al, with both studies observing delayed motor development in 33% of patients, both MPHD and ICCH.

Limitations of our study include its retrospective nature, changes in newborn screening strategies over the study period, and potential underdiagnosis due to incomplete enrollment.

Neurodevelopmental assessment in the study relied on evaluations provided by developmental pediatricians or pediatric neurologists, as well as interventions conducted within neurologic and child development departments. Not all children underwent uniform validated psychological or neurological assessments.

## Conclusions

Our study sheds light on the challenges in the diagnosis and management of CCH. While the prevalence of neurologic sequelae was similar between early and late diagnosis, the overall high prevalence of neurodevelopmental sequelae in our cohort underscores the critical importance of early detection and treatment of CCH to prevent long-term complications. Further studies should be conducted to evaluate the contributions of delayed diagnosis to the neurologic prognosis of newborns with CCH.

## Data Availability

The datasets generated and/or analyzed during the current study are available from the corresponding author upon reasonable request.

## Abbreviations

ACTH: adrenocorticotropic hormone
CCH: central congenital hypothyroidism
CH: congenital hypothyroidism
FT4: free thyroxine
ICCH: isolated central congenital hypothyroidism
LH: luteinizing hormone
LT4: levothyroxine
MPHD: multiple pituitary hormone deficiencies
MRI: magnetic resonance imaging
NBS: newborn screening
PSIS: pituitary stalk interruption syndrome
TBG: thyroxine-binding globulin
TSH: thyrotropin (thyroid-stimulating hormone)
TT4: total thyroxine
